# Positions 299 and 302 of the GerAA subunit are important for function of the GerA spore germination receptor in *Bacillus subtilis*

**DOI:** 10.1371/journal.pone.0198561

**Published:** 2018-06-01

**Authors:** Anna Grela, Inga Jamrożek, Marta Hubisz, Adam Iwanicki, Krzysztof Hinc, Rajmund Kaźmierkiewicz, Michał Obuchowski

**Affiliations:** 1 Laboratory of Molecular Bacteriology, Department of Medical Biotechnology, Intercollegiate Faculty of Biotechnology UG & MUG, University of Gdańsk, Gdańsk, Poland; 2 Laboratory of Biomolecular Systems Simulations, Intercollegiate Faculty of Biotechnology UG & MUG, University of Gdańsk, Gdańsk, Poland; 3 Laboratory of Molecular Bacteriology, Department of Medical Biotechnology, Intercollegiate Faculty of Biotechnology UG & MUG, Medical University of Gdańsk, Gdańsk, Poland; University of Connecticut, UNITED STATES

## Abstract

*Bacillus subtilis*, as a model spore-forming Gram-positive bacterium, has been extensively used for spore germination research. Within this field, nutrient-dependent germination with specific germinant receptors (GerA, responding to L-alanine or L-valine; GerB and GerK, acting together to start spore germination process in response to AGFK) has been the most studied. There are three different variants of the GerAA subunit (299T/302S, 299A/302P, 299A/302S) of the GerA germination receptor present in *B*. *subtilis* subs. *subtilis* laboratory strains. According to our research, the 299A/302P one, unlike the others, interferes with the spore’s ability to germinate in L-alanine as assessed by the measurement of DPA release upon stimulation with the germinant. Multiple genetic manipulations described in this work followed by spore germination tests, together with secondary structure predictions led us to the following conclusions. First, position 302 of GerAA protein is crucial in terms of GerA germination receptor functionality; a proline residue at this position renders the GerA receptor non-functional, most probably due to a change in the protein secondary structure. Second, the 302P GerAA variant has most probably an impaired affinity to other components of GerA receptor. Together, these may explain the loss of GerA receptor’s function. Analysis of the GerAA protein should get us closer to understanding the mechanism of GerA receptor function.

## Introduction

*Bacillus subtilis*, as a model Gram-positive bacterium, has been extensively used in genetic and behavioural studies, focusing on many diverse physiological aspects common for the spore-forming species. Currently, *B*. *subtilis* 168, subsp. *subtilis*, and its derivatives, including PY79 and PS832, are used for research in a variety of laboratories worldwide [1]. *B*. *subtilis* 168, a highly transformable tryptophan auxotroph, was derived from the Marburg strain by X-ray mutagenesis [2] and since that time multiple studies have been performed on this strain by different research groups worldwide, leading to the appearance of spontaneous mutations. It has been indeed reported that there are 31 single-base differences between two isolates of 168, BGSC 1A1 and BGSC 1A700, deposited in the *Bacillus* Genetic Stock Center [3]. At the beginning of the genetic studies on *B*. *subtilis*, many of the new strains were constructed using *B*. *subtilis* subsp. *spizizenii* W23 and its derivatives as DNA donors which led to the appearance of 168-W23 hybrids [1]. Out of two mentioned 168 derivative strains, genomic DNA of PY79 is the most divergent from the one of their last common ancestor– 168. It contains the sequences identical to that of W23 in the surroundings of *trpC* and *sacA* loci, four large and some smaller deletions as well as 81 SNPs in comparison to 168 genome. All of the genomic differences between 168, PY79 and PS832, together with the most possible construction flow of all of these strains are described and discussed in detail elsewhere [1].

During the project of systematic function analysis of *B*. *subtilis* genes funded by the EU under 4^th^ Framework (BIO4950278), which aimed to determine the functions of unknown *B*. *subtilis* 168 genes identified during sequencing of its genome [4], phenotypic differences between the background strains used for the analyses were found. However, the genetics underlying those phenotypic changes were not studied. Herein we report for the first time that there are two physiologically distinct variants of 168 in terms of L-alanine-dependent spore germination caused by two single nucleotide changes in the *gerAA* gene.

Germination is a physiological process of all spore-forming species which leads to the return of metabolically dormant endospores to the vegetative growth in the presence of specific nutrient and non-nutrient stimuli, the former being the most important in terms of spore germination in nature [5, 6]. In *B*. *subtilis*, three germination receptors (GRs) in the spore’s inner membrane are involved in the response to different nutrient germinants [7, 8]. While GerA-dependent germination is induced by L-alanine or L-valine alone, the mixture of AGFK (L-asparagine, glucose, fructose and K^+^ ions) needs the presence of both GerB and GerK GRs to trigger germination [9]. All GRs consist of three subunits (A, B and C) encoded by polycistronic *gerA* operon homologs, *gerA*, *gerB* and *gerK*, expressed during sporulation in a σ^G^-dependent manner. Two other *gerA* operon homologs, *yndDEF* and *yfkQRT*, are also present in the *B*. *subtilis* 168 genome, as revealed by whole-genome sequencing, however they have not been yet reported to encode any active GR.

GerA is probably the most studied GR of all. Recent research on laboratory strains 168 (subsp. *subtilis*) and W23 (subsp. *spizizeni*), and on several other recently isolated environmental strains of both subspecies, has shown the conservation of GerA receptor’s function among those strains [10]. The GerA GR comprises two hypothetical transmembrane proteins, GerAA (BSU33050) and GerAB (BSU33060), and a predicted lipoprotein, GerAC (BSU33070). GerAA is believed to have a central hydrophobic domain with 4 to 6 predicted membrane-spanning alpha-helices and two hydrophilic domains, a large N-terminal and substantially smaller C-terminal domain [11, 12]. As the GerAA structure has not been solved, it is impossible to define the role of individual GerAA residues, which may range from the assembly of the functional receptor to the binding of germinant. In a recent study several laboratory-constructed GerAA mutants with severe defects in L-alanine-triggered germination were described [13]. Some of those cause the loss of GerAC protein from the spore, thus suggesting that mutations in *gerAA*, resulting in a change of a single residue, may impact on either GerAA alone or the whole GerA receptor [13]. Functional *in vivo* analysis of different *gerAA* mutants seems to be, for now, the only way to assess the importance of any particular residue in GerA-dependent germination.

Our research shows for the first time the impact of different *gerAA* alleles, naturally occurring in *B*. *subtilis* subsp. *subtilis*, on endospore germination. Spores of one of two studied *B*. *subtilis* 168 isolates, called here 168F (of French origin), are entirely unresponsive to L-alanine. Analysis of spore germination kinetics in different *B*. *subtilis* strains, combined with secondary structure prediction for GerAA, including detection of likely transmembrane segments, suggest a plausible explanation for the L-alanine-dependent germination defect of 168F spores.

Differences in *gerAA*, as well as other differences between the genomes of *B*. *subtilis* 168 variants/derivatives presented in this work and elsewhere [1], should be considered while performing experiments on 168 mutants coming from different research groups, and while comparing data described in the literature.

## Results & discussion

### L-alanine-dependent germination of GerAA variants

#### Three GerAA variants in different *B*. *subtilis* strains

**Fig 1:**
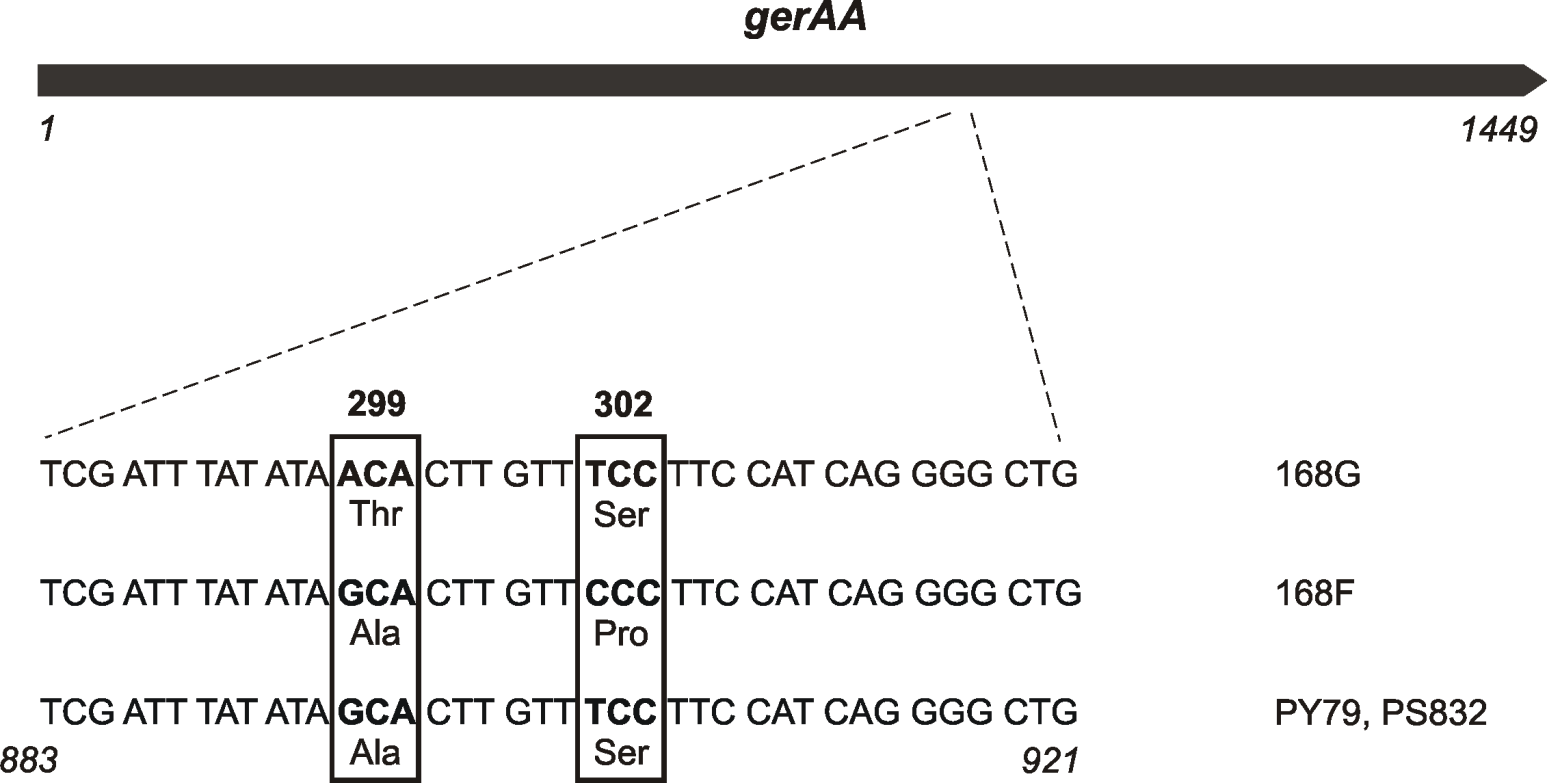
Differences in the *gerAA* gene among different *B*. *subtilis* laboratory strains. Magnification of the 883–921 nucleotide region of *gerAA* variants present in the depicted laboratory *B*. *subtilis* strains, listed on the right, is shown. Codons with the single nucleotide polymorphisms, presented in bold, are boxed together with the altered amino acid residues at position 299 and 302 of GerAA amino acid chain.

In the project of systematic function analysis of *Bacillus subtilis* genes (BIO4950278), 1146 genes of unknown function were disrupted and resulting mutants were characterized by analyzing their growth properties, activity of reporter gene as well as systematic determination of phenotype. The results of analysis, along with genomic data, were deposited in the Micado database [14]. One of the phenotypic features analyzed in the project were spore germination properties. Germination tests were based on reduction of the tetrazolium salt to the red formazan resulting in pink or red coloring of spore-containing colonies, depending on the rate of germination. It was reported that in some laboratories participating in the project the reference strain differed in test results, forming white colonies instead of red. To identify the cause of this phenomenon, we selected two 168 strains from different laboratories, called 168F (origin of France) and 168G (origin of Germany) (S2 Table) that differed in their ability to resume oxidative metabolism in the tetrazolium germination test. While colonies of 168G strain turned red, 168F colonies remained white, suggesting a defect in the germination process. Upon verification of germination properties, chromosomal DNA of both strains was isolated and sent for whole-genome sequencing. The short reads obtained were assembled based on the reference genome sequence of 168 strain (NC_000964.3) and consensus sequences annotated, and deposited with accession numbers CP016852.1 (168G) and CP010052.1 (168F) at GenBank. In the next step, we performed genome alignment, including also the genome sequences of PS832 (CP010053.1) and PY79 (NC_022898.1) strains, which are variants of wild-type *B*. *subtilis* 168 popular in numerous laboratories. Major differences between 168, PS832 and PY79 genomes were described previously in detail by [1]. The full list of the differences between 168G and 168F genomes are listed in S1 and S2 Tables. According to the genome alignment, the most interesting changes, regarding the phenotype of altered spore germination are located in positions 895 and 904 of the *gerAA* gene. These changes result in the following alterations of amino acid sequence of GerAA protein: 299T/302S for 168G, 299A/302P for 168F, and 299A/302S for PY79 and PS832 (Fig 1).

#### Spores of different *B*. *subtilis* laboratory strains respond differently to L-alanine

**Fig 2:**
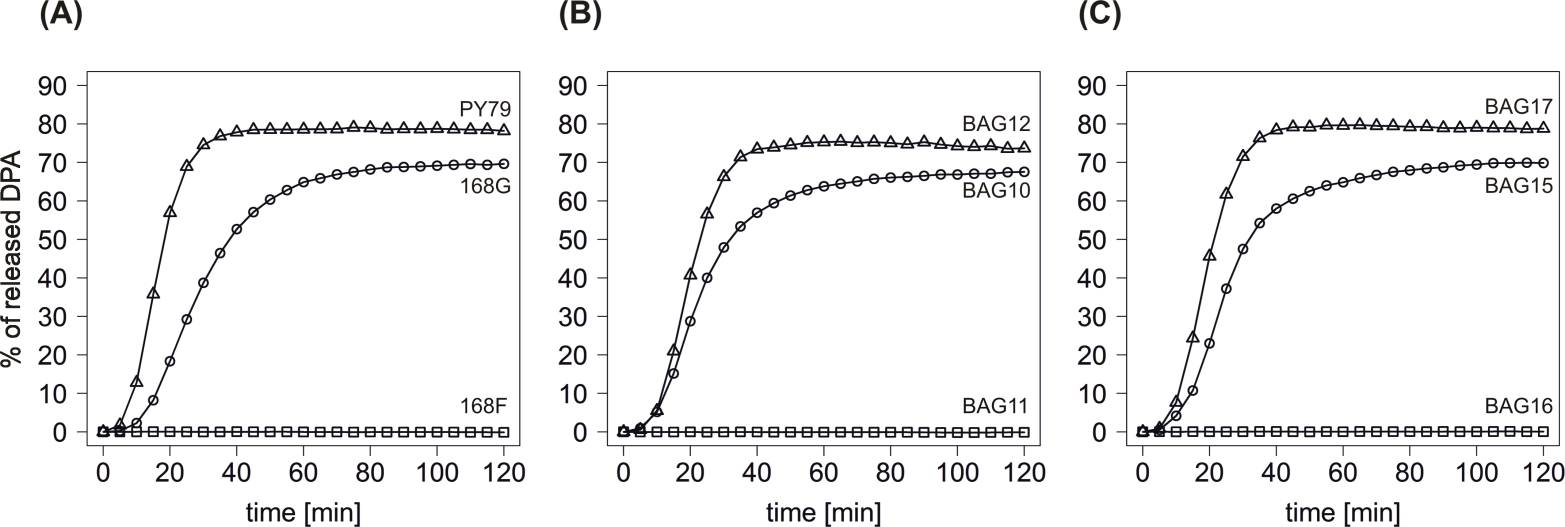
DPA release from germinating spores of different *B*. *subtilis gerAA* variants. Germination triggered by 10mM L-alanine was analyzed by measurement of DPA release. (A) 168G with 299T/302S *gerAA* (circles), 168F with 299A/302P *gerAA* (squares) and PY79 with 299A/302S *gerAA* variant (triangles). (B) Complementation of 168F (299A/302P *gerAA*) with: 299T/302S *gerAA* (BAG10, circles), 299A/302P *gerAA* (BAG11, squares) and 299A/302S *gerAA* (BAG12, triangles). (C) BAG15 (circles), BAG16 (squares) and BAG17 (triangles) with one *gerAA* allele at *amyE*: 299T/302S, 299A/302P and 299A/302S, respectively.

Changes in the amino acid sequence of GerAA, listed above, in the selected laboratory strains suggested that there might be some differences in GerA-dependent germination between these [13]. Indeed, spore germination analysis with L-alanine as the sole germinant showed that: i) 168F spores with 299A/302P GerAA are unable to germinate in the presence of 10mM or 100mM L-alanine (Fig 2A, Table 1), concentration far exceeding the saturating one needed for GerA-dependent germination in different laboratory and wild type *B*. *subtilis* strains [10]; ii) 168G and PY79 spores with 299T/302S and 299A/302S GerAA, respectively, germinate in the presence of 10mM L-alanine; however the kinetics of spore germination differ between these (Fig 2A, Table 1). The maximum rate of germination of PY79 spores was more than twice as high as in the case of 168G spores. Also, the microlag time (the time between the exposure of the spores to the germinant and detection of the DPA) of PY79 spores was slightly shorter than the one of 168G spores. In concordance with all that, the time in which half-maximal amount of DPA was released from PY79 spores during germination in 10mM L-alanine was just over half that of 168G spores. Moreover, the maximum amount of DPA released from 168G and PY79 spores, which also correlated with the percentage of spores becoming phase-dark during GerA-dependent germination assay, differed by 10% and was higher in the case of PY79 spores (Table 1). The latter is consistent with data presented lately by [15]. In this as well as all the other germination experiments described in this work, 70 and 80% of DPA released from spores correlated each time with around 91 and 99,9% of phase-dark spores, respectively. The phase-dark spores count was only used to confirm the DPA release measurements and, as the less precise equivalent to the fluorescence detection, it was not included in the descriptions of spore germination kinetics.

**Table 1:** Parameters of GerA-dependent spore germination of various *B*. *subtilis* strains.

*gerAA* variant at *gerA^a^*	*gerAA* variant at *amyE^a^*	Strain^*b*^	Max. % of DPA released from the spores ± SD	Max. rate of germination [% of released DPA / min] ± SD	DR50 [min]^*d*^ ± SD
299T/302S	-	**168G**	70 ± 8.2	2.4 ± 0.30	28 ± 3.8
299A/302P	-	**168F**	0 ± 0.1	-	-
299A/302P	-	**168F** ^*c*^	0 ± 0.1	-	-
299A/302S	-	**PY79**	80 ± 6.3	5.3 ± 0.76	16 ± 2.4
299A/302P	299T/302S	**BAG10**	68 ± 5.7	2.8 ± 0.19	22 ± 2.5
299A/302P	299A/302P	**BAG11**	0 ± 0.1	-	-
299A/302P	299A/302S	**BAG12**	76 ± 4.8	4.1 ± 0.20	19 ± 2.5
-	299T/302S	**BAG15**	71 ± 5.4	2.9 ± 0.88	25 ± 2.5
-	299A/302P	**BAG16**	0 ± 0.3	-	-
-	299A/302S	**BAG17**	81 ± 11.7	4.3 ± 0.75	19 ± 1.9
-	299A/302A	**BAG18**	65 ± 5.9	3.2 ± 0.46	19 ± 1.9
-	299A/302G	**BAG20**	73 ± 7.7	3.1 ± 0.53	25 ± 1.6
299T/302S	↑299T/302S	**BAG21**	72 ± 8.7	3.9 ± 0.90	19 ± 4.0
299T/302S	↑299A/302P	**BAG22**	59 ± 4.1	2.1 ± 0.20	27 ± 3.1
299A/302P	↑299T/302S	**BAG23**	73 ± 6.7	4.0 ± 0.34	20 ± 3.5
299A/302P	↑299A/302P	**BAG24**	0 ± 0.2	-	-
299A/302P	↑299A/302S	**BAG26**	78 ± 9.9	5.9 ± 0.70	13 ± 0.7
299A/302S	↑299A/302S	**BAG27**	74 ± 5.2	6.0 ± 0.65	13 ± 1.1
299A/302S	↑299A/302P	**BAG29**	67 ± 4.8	3.2 ± 0.20	18 ± 0.7
-	299T/302P	**BAG30**	0 ± 0.1	-	-
-	299T/302S	**BAG31**	68 ± 5.7	2.1 ± 0.34	30 ± 3.6
-	299A/302P	**BAG32**	0 ± 0.1	-	-
-	299A/302S	**BAG33**	77 ± 5.2	5.6 ± 2.28	16 ± 6.1

^*a*^ *gerAA* variants differ in the sequence of two codons resulting in the alteration at positions 299 and 302 of GerAA amino acid residue chain; *gerAA* in the depicted loci were expressed from P_*gerA*_, except for *gerAA* variants with an upward pointing arrow (↑) where *gerAA* was placed under the control of strong *sspB* promoter.

^*b*^ Germination was triggered by 10mM L-alanine solution unless stated differently.

^*c*^ Spore germination was triggered by 100mM L-alanine solution.

^*d*^ DR50 –time, in minutes, in which half-maximal amount of DPA was released during spore germination assays.

#### Three GerAA variants present in different *B*. *subtilis* laboratory strains are responsible for the altered spore germination in response to L-alanine

Complementation of 168F with the *gerAA* allele from 168G (BAG10) and PY79 (BAG12) at *amyE* led to the change of germination phenotype of 168F to the one observed in 168G and PY79 (Fig 2B), respectively. Even though all kinetic parameters differed slightly between germinating 168G and BAG10 as well as PY79 and BAG12 spores, the percentages of DPA released from spores upon completing the assay, which reflected the percentage of phase-dark spores, were essentially the same in the depicted pairs (Table 1). The difference in the maximum rate of germination was still clear for BAG10 and BAG12 as it was for L-alanine-responsive wild-type spores. However, we did not observe this time any visible difference in microlag times. As expected, BAG11, with two copies of 299A/302P *gerAA*, did not germinate in 10mM L-alanine (Fig 2B).

The full-length *gerA* operon was next deleted from the 168G chromosome and complemented separately with different *gerAA* alleles: 299T/302S (BAG15), 299A/302P (BAG16) and 299A/302S (BAG17) at *amyE* and *gerAB*, *gerAC* at *thrC*, all of them under the control of native P_*gerA*_ promoter (Fig 2C). Similar constructs were prepared and tested in the 168F genetic background (BAG31, BAG32 and BAG33; Table 1). Results demonstrate that the germination phenotype of 168G, 168F and PY79 spores with different *gerAA* variants is background-independent. Indeed, all of the L-alanine-responsive spores with the same *gerAA* allele (168G, BAG10, BAG15, BAG31 with 299T/302S or PY79, BAG12, BAG17, BAG33 with 299A/302S *gerAA*) were germinating at the very similar speed and released similar amounts of DPA within the tested time frame, the latter reflecting essentially the same percentages of phase-dark spores in the tested spore suspensions. Minor differences within the groups of spores with the same GerAA variant might have been observed if lower concentrations of L-alanine had been tested, especially in case of strains with two *gerAA* alleles (BAG10 and BAG12), however, it was not checked. The most comparable between the groups differing in all the mentioned kinetic parameters was the microlag. Any of the spores with 299A/302P GerAA only was able to germinate in the presence of L-alanine. Based on the above, 299A/302P GerAA is fully non-functional in terms of L-alanine-dependent germination at saturating concentrations of L-alanine, while GerA GR formed by 299T/302S or 299A/302S GerAA can respond to this nutrient germinant, however that response is detectably different.

### Secondary structure alterations between 302S and 302P GerAA variants can explain altered GerA-dependent spore germination response

One possible reason for the alteration of spore germination phenotype between 302S and 302P GerAA could be a change in protein structure. This hypothesis is supported by the fact that proline has the lowest affinity among all amino acids for helix formation due to its specific structure [16]. Lack of amide proton, which forms the characteristic hydrogen bond within the helix, as well as the presence of the ring formed by the backbone and side chain result in that proline cannot be fully incorporated into an α-helix. Prediction of the secondary structure of the GerAA protein indicated that residues at 299 and 302, which are both the main focus of this work, are likely to be located in a long α-helical structure, consisting of residues 274–302 (S1 Fig). Moreover, a substantial part of this helix was predicted by different transmembrane fragment prediction programs to form a membrane spanning fragment: i) TMAP–residues 280–308; ii) TMPred–residues 284–300; iii) TMHMM–residues 279–301. These predictions are consistent with the earlier work [8], which suggests that GerAA protein is an integral membrane protein. All GerAA transmembrane fragments predicted by different servers are listed in S3 Table.

Taking all of the above together, it is highly probable that the change in position 302 of GerAA from serine to proline disrupts GerAA activity, and in consequence the activity of the whole GR, due to structural reasons. What is more, as the 274–302 helix would contain a membrane spanning region, even the slightest change in the structure or length of this helix may influence the manner in which GerAA protein is embedded into the inner spore membrane. This disturbance may also account for the observed differences in GerA GR functionality.

### Attempts to elucidate the impact of GerAA residue 302 on GerA GR functioning

Analysis of spore germination kinetics described so far in this work together with secondary structure predictions suggested that position 302 of GerAA has a major impact on GerA GR functionality. However, on the basis of all the results described above we could not exclude that a change at 299 from A to T in the 299A/302P GerAA variant might influence the germination phenotype of non-L-alanine responding spores (168F, BAG16, BAG32). To test this and cover all of the possible combinations of naturally occurring amino acid residues at positions 299 and 302 of GerAA at the same time, we also constructed a strain with 299T/302P GerAA (BAG30). As expected, the GerA GR containing 299T/302P GerAA was not functional in terms of L-alanine-dependent germination (Table 1) proving that the differences in GerA functionality are strictly dependent on residue 302.

#### Effect of residue substitution at position 302

**Fig 3:**
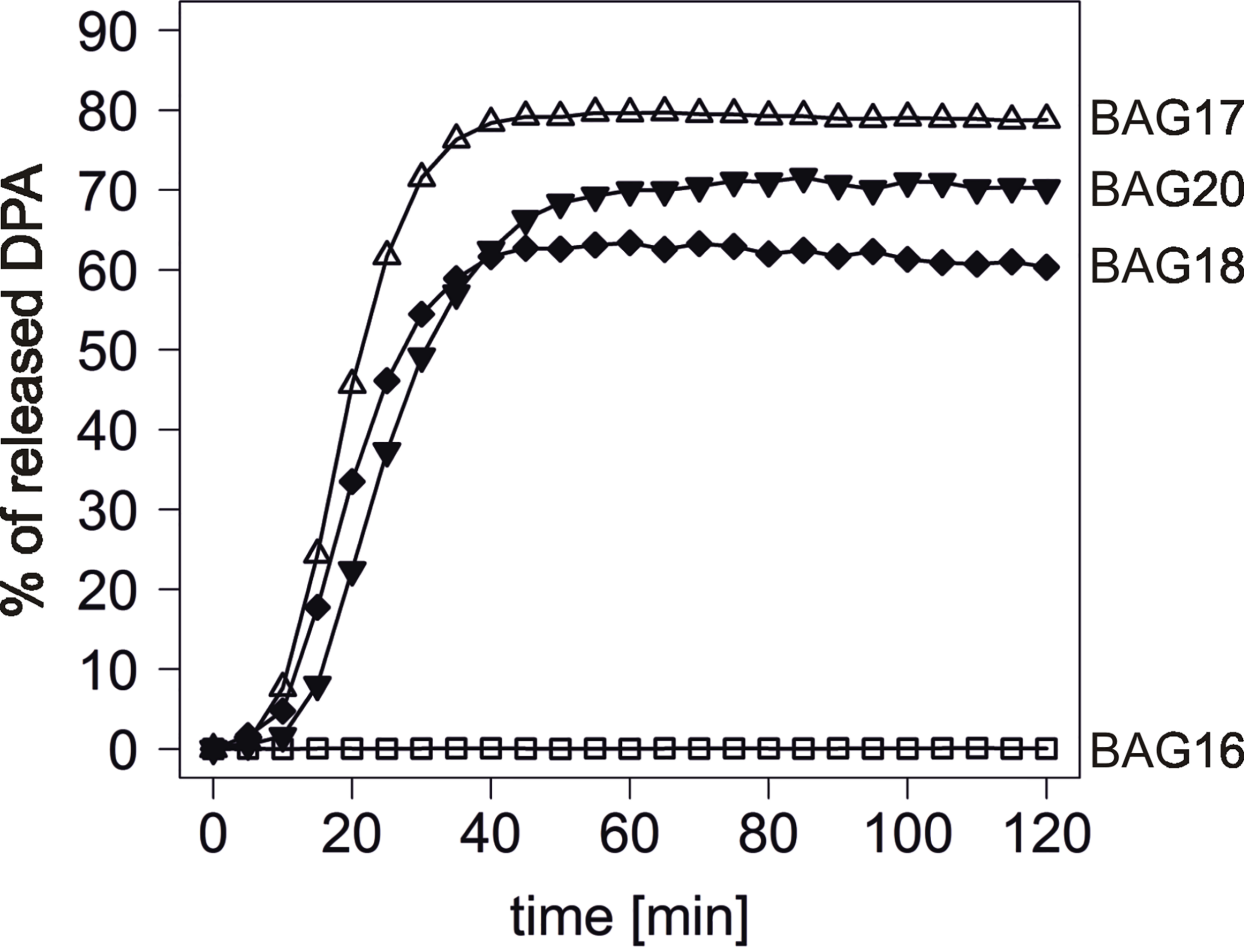
Spore germination of the strains with different *gerAA* alleles. Germination triggered by 10mM L-alanine was analyzed by measurement of DPA released from the spore core. BAG16, 299A/302P *gerAA* (open squares); BAG17, 299A/302S (open triangles); BAG18, 299A/302A *gerAA* (filled diamonds); BAG20, 299A/302G *gerAA* (filled point-down triangles).

Taking into account the possible influence of proline on proteins’ α-helical secondary structures, we tested whether other changes in position 302 might change the functionality of the GerA GR, by altering the secondary structure of GerAA protein. We therefore constructed two *B*. *subtilis* strains carrying new versions of GerAA: 299A/302A (BAG18) and 299A/302G GerAA (BAG20). Having expected that glycine properties in terms of alpha-helix disruption might be similar to those of proline [16], we predicted that 168F and BAG20 might have the same L-alanine germination phenotype, while BAG18 spores would probably be able to germinate in this nutrient germinant. In fact, spores of both strains were able to germinate in 10mM L-alanine solution with kinetic parameters and microlag times in between the ones obtained for 168G and PY79 spores, implying no significant differences in GerA GR functionality in these (Fig 3, Table 1). The GerAA germination defect in 168F therefore is because of the exceptional proline conformational properties in comparison to other amino acid residues, including glycine. As mentioned before, minor effects of different amino acid substitutions at 302 of GerAA, in particular the sensitivity of the spores to L-alanine, could have been detected if more limiting levels of the germinant had been used in the germination assays as it was analyzed for a number of GerAA mutants elsewhere [13]. However, as the main goal of the study was to check the general phenotype of the spores with different 302 GerAA substitutions, the secondary effects of these were not studied here in detail.

Spore germination analysis of the strains varying in positions 299 and 302 of GerAA described so far in this work led us to the following conclusions: i) the alteration in 299 changes the kinetics of germination in case of L-alanine responsive strains (168G, PY79, BAG15, BAG17, BAG31, BAG33) (Fig 2A and 2C, Table 1); ii) change in position 302 of GerAA from serine, alanine or glycine to proline (Table 1) has a major impact on GerA GR functionality, most probably due to structural reasons.

#### 299A/302P GerAA has lowered affinity to the other subunits of GerA GR

In the experiments described above we performed genetic complementation of 168F strain (unable to respond to the presence of L-alanine) with *gerAA* alleles from 168G or PY79. The resulting strains (BAG10 and BAG12, respectively) showed the phenotype of *gerAA* donors, i.e., 168G or PY79, as assessed by the measurement of the total amount of DPA released from the spores (Fig 2, Table 1). Summarizing these results, when one of the functional alleles (299T/302S GerAA from 168G or 299A/302S GerAA from PY79) was present in the spore at the same level with non-functional allele (299A/302P GerAA from 168F) we observed a full dominance of the functional GerAA variant over the non-functional one. Following the idea that S302P most probably causes a structure alteration of the GerAA protein, we wanted to check whether this structural change does or does not influence the assembly/functioning of the whole GerA GR. 168G (299T/302S GerAA) and PY79 (299A/302S GerAA) were thus complemented with an overexpressed non-functional 299A/302P *gerAA* allele from P_*sspB*_ promoter at *amyE* (BAG22 and BAG29, respectively) to check how it would influence the L-alanine germination rate and/or kinetics of the background strain. According to the previous work, overexpression of GerAA (when *gerAA* was under control of P_*sspB*_) does not seem to influence the sporulation efficiency as it was observed in case of whole *gerA* operon transcription from P_*sspB*_ [17]. It was also described earlier that levels of GerBA (BSU35800), a GerAA homolog, in spores with the *gerB* operon under the control of the strong, σ^G^-dependent P_*sspB*_ were ~200- to ~500-fold higher than in the wild type strains [17, 18]. In other work, different *gerAC* alleles were successfully overexpressed from P_*sspB*_ at *amyE* as proved by spore germination analysis. Moreover, in the same study excess of a mutant, non-functional GerAC in spores with the normal levels of wild-type GerAC led to a significant decrease of L-alanine-dependent germination in comparison to the background strain [19]. Basing on the latest report, in which GerAA and GerAC levels were accounted for around 1100 molecules of each subunit per spore in physiological conditions [20], and the levels of GR subunits in spores, described above, when their genes were placed under strong *sspB* promoter, we predicted that the overexpression of a non-functional GerAA subunit might lead to a significant decrease of GerA functionality as was shown before for GerAC [19].

The control strains (BAG23 and BAG26), with two *gerAA* alleles, *gerAA* 299A/302P under P_*gerA*_ at *gerA* and 299T/302S or 299A/302S *gerAA*, respectively, transcribed from P_*sspB*_ at *amyE*, were able to germinate in 10mM L-alanine (Table 1). The maximum rates of germination of analyzed strains were higher, though, than in: i) the corresponding (in terms of the presence of functional GerAA variant) wild-type strains, 168G and PY79, respectively, or ii) spores of the other strains described in this work, carrying *gerAA* under P_*gerA*_ at *amyE* (BAG10, BAG15, BAG31 and BAG12, BAG17, BAG33, respectively) (Table 1). In the case of BAG21 and BAG27 with the same two *gerAA* alleles encoding functional variants of GerAA (either 299T/302S or 299A/302S GerAA, respectively) under P_*gerA*_ at *gerA* and P_*sspB*_ at *amyE* we observed essentially the same germination kinetics as for BAG23 and BAG26, respectively. Considering that GerA GR is formed out of equal number of A, B and C subunits, which is suggested by a single transcription unit of GerA GR genes, in BAG21, BAG23, BAG26 and BAG27 the level of B and C subunit of GerA receptor should be theoretically a limiting factor for GerA GR assembly and consequently GerA-dependent germination. Therefore, it might be expected that highly increased amounts of GerAA should have generally no effect on germination in L-alanine solution. In contrast, our results and those of other authors are consistent in terms of an increase in GerA-dependent germination if the number of one of the GerA subunits was strongly elevated [19]. However, this correlation was much stronger in the work of Igarashi and co-workers. This may be due to the fact that in that study the authors used the strains lacking GerB and GerK GRs which might have strengthened the germination phenotype of the analyzed strains. The other possibility is that the importance of different GerA subunits in GerA GR assembly is different. All in all, data suggest that the overall activity of GR receptors increases somewhat if one of the GerA subunits is present in excess. Our results proved that the designed constructs, with *gerAA* under P_*sspB*_, had led to the proper synthesis of GerAA. As expected, spores of BAG24 with two copies of 299A/302P *gerAA*, one under P_*gerA*_ at *gerA* and the other P_*sspB*_ at *amyE* (high levels of non-functional GerAA protein) were unable to germinate in the presence of L-alanine (Table 1).

**Fig 4:**
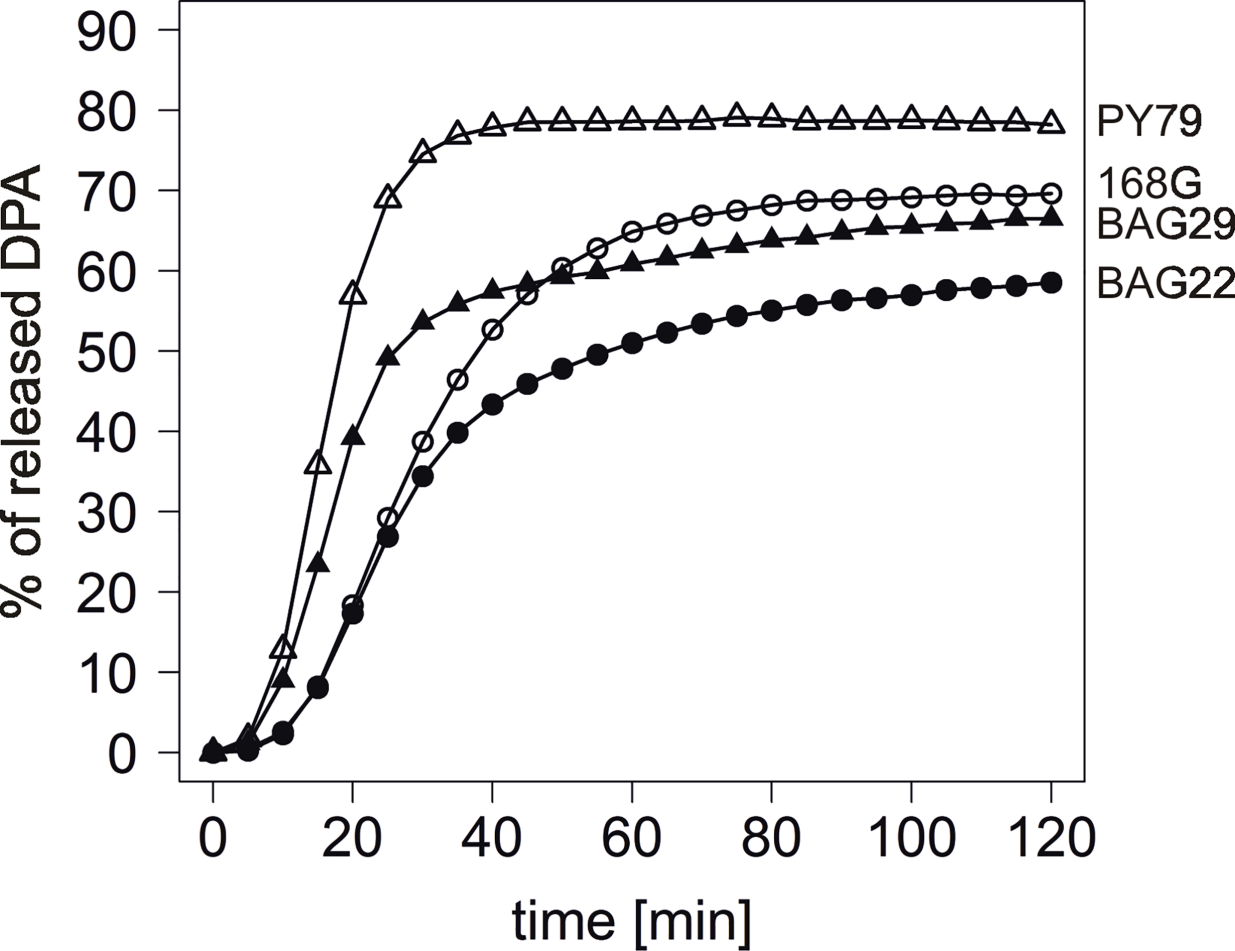
Overproduction of non-functional GerAA variant in wild-type *B*. *subtilis* strains–spore germination analysis. Spore germination triggered by 10mM L-alanine was analyzed by DPA spore core content release measurement. Open symbols: 168G (circles); PY79 (triangles). Filled symbols: 168G with additional *gerAA* (299A/302P) under P_*sspB*_ at *amyE* (BAG22, circles); PY79 with additional *gerAA* (299A/302P) under P_*sspB*_ at *amyE* (BAG29, triangles).

Germination of BAG22 (299T/302S GerAA + ↑299A/302P GerAA) and BAG29 (299A/302S GerAA + ↑299A/302P GerAA) in 10mM L-alanine solution, though showing a very similar microlag, was lower than in the background wild-type strains, 168G and PY79, respectively (Fig 4, Table 1). However, the changes in the maximum rate of germination and the maximum amount of DPA released from the spores, corresponding to the amount of spores in the tested population which had undergone the germination process, were not as substantial as would be expected if the defective subunit was competing entirely efficiently for assembly. On the basis of this we have thus speculated that 299A/302P GerAA has lower affinity to other GerA GR subunits in comparison to 299T/302S or 299A/302S GerAA. As a 1:1 ratio of functional (299T/302S or 299A/302S) and non-functional GerAA (299A/302P) subunit present in the spore does not change the functionality of the whole GerA GR in comparison to the ones present in 168G or PY79 spores (Fig 2, Table 1), on the basis of our results we may imply that the non-functional 299A/302P GerAA can bind to other GerA GR components when this ratio is around 1:100 or higher. However, even if the assembly of the GerA GR occurs when the level of 299A/302P GerAA in the spore is highly elevated, this GerAA subunit remains still non-functional in terms of GerA-dependent germination. Indeed, in the case of BAG24 spores (299A/302P + ↑299A/302P GerAA) we did not observe any improvement in spores’ response to L-alanine as the sole germinant (Table 1). Taking all of the above together, we suggest that in the case of 299A/302P GerAA variant we face a superposition of two effects: the impairment of 299A/302P GerAA in joining the GerA GR complex as well as the loss of protein’s function. Both can be a result of the structural alterations between functional (299T/302S and 299A/302S) and non-functional (299A/302P) naturally occurring GerAA variants.

## Conclusions

To summarize the findings described in this work we have shown that position 299 of GerAA influences the efficiency and kinetics of GerA-dependent germination. On the other hand, the presence of proline residue at position 302 of the GerAA subunit renders the GerA GR non-functional and lowers affinity of this subunit to other components of the receptor, most probably due to structural changes.

## Materials and methods

### Genome sequencing and sequence comparison

The chromosomal DNA of *B*. *subtilis* 168F, 168G and PS832 were isolated and send for NGS at BaseClear company (www.baseclear.com). Obtained short reads were assembled by BaseClear based on the reference genome sequence of 168 strain (accession number NC_000964.3). Complete genome sequences were deposited at GenBank (accession numbers CP016852.1, CP010052.1 and CP010053.1 for 168G, 168F and PS832, respectively). Comparison of genomic sequences was done in Mauve ver. 2.3.1. [21].

### Construction of plasmids and strains

All *B*. *subtilis* strains used in this study are listed in Table 2. *B*. *subtilis* 168F strains carrying a second allele of *gerAA* at *amyE* (BAG10 to 12) were constructed as follows. Full-length *gerAA* gene sequence with its natural promoter was PCR amplified from the following chromosomal templates: *B*. *subtilis* 168G, *B*. *subtilis* 168F and *B*. *subtilis* PY79, carrying different naturally occurring *gerAA* variants: 299T/302S, 299A/302P and 299A/302S, respectively. The primers used contained an *Eco*RI (gerAA-Eco upstream primer) and *Bam*HI (gerAA-Bam downstream primer) sites enabling cloning PCR products into pDL integration vector [22]. Resulting vectors (pAG100, pAG101 and pAG102, respectively) were linearized and transformed into *B*. *subtilis* 168F, selecting for chloramphenicol resistance, generating BAG10, BAG11 and BAG12, respectively. Chromosomal integration was confirmed by PCR method with the primers upstream and downstream to *amyE*.

**Table 2:** List of strains used or constructed during the study.

Strain	Genotype^*a*^	Source or construction^*b*^
168G	*trpC2*	From M. Hecker
168F	*trpC2*	From S. Seror
PY79	Prototroph SPβ^s^	From E. Ricca
PS832	Wild-type prototroph^*c*^	From P. Setlow
BAG07	Δ*gerA*	BAG09 → 168F
BAG08	Δ*gerA thrC*:: *gerAB/C*	pAG110 → BAG07
BAG09	Δ*gerA*	pNC-ilaR → PY79
BAG10	*amyE*:: *gerAA* (299T/302S)	pAG100 → 168F
BAG11	*amyE*:: *gerAA* (299A/302P)	pAG101 → 168F
BAG12	*amyE*:: *gerAA* (299A/302S)	pAG102 → 168F
BAG13	Δ*gerA*	BAG09 → 168G
BAG14	Δ*gerA thrC*:: *gerAB/C*	pAG110 → BAG13
BAG15	Δ*gerA thrC*:: *gerAB/C amyE*:: *gerAA* (299T/302S)	pAG100 → BAG14
BAG16	Δ*gerA thrC*:: *gerAB/C amyE*:: *gerAA* (299A/302P)	pAG101 → BAG14
BAG17	Δ*gerA thrC*:: *gerAB/C amyE*:: *gerAA* (299A/302S)	pAG102 → BAG14
BAG18	Δ*gerA thrC*:: *gerAB/C amyE*:: *gerAA* (299A/302A)	pAG103 → BAG14
BAG20	Δ*gerA thrC*:: *gerAB/C amyE*:: *gerAA* (299A/302G)	pAG105 → BAG14
BAG21	*amyE*::P_*sspB*_*gerAA* (299T/302S)	pAG112 → 168G
BAG22	*amyE*::P_*sspB*_*gerAA* (299A/302P)	pAG113 → 168G
BAG23	*amyE*::P_*sspB*_*gerAA* (299T/302S)	pAG112 → 168F
BAG24	*amyE*::P_*sspB*_*gerAA* (299A/302P)	pAG113 → 168F
BAG26	*amyE*::P_*sspB*_*gerAA* (299A/302S)	pAG114 → 168F
BAG27	*amyE*::P_*sspB*_*gerAA* (299A/302S)	pAG114 → PY79
BAG29	*amyE*::P_*sspB*_*gerAA* (299A/302P)	pAG113 → PY79
BAG30	Δ*gerA thrC*::*gerAB/C amyE*::*gerAA* (299T/302P)	pAG115 → BAG14
BAG31	Δ*gerA thrC*::*gerAB/C amyE*::*gerAA* (299T/302S)	pAG100 → BAG08
BAG32	Δ*gerA thrC*::*gerAB/C amyE*::*gerAA* (299A/302P)	pAG101 → BAG08
BAG33	Δ*gerA thrC*::*gerAB/C amyE*::*gerAA* (299A/302S)	pAG102 → BAG08

^*a*^ In round brackets variant of *gerAA* introduced to *B*. *subtilis* chromosome is described (299X and 302Z correspond to X and Z amino acid residue in GerAA amino acid chain at position 299 and 302, respectively).

^*b*^ Arrows represent the transformation event of the strain pointed on the right side of the arrow with the plasmid or chromosomal DNA depicted on its left side. Construction of all listed plasmids is described in ‘Construction of plasmids and strains’ section.

^*c*^ a prototrophic laboratory derivative of strain 168.

To obtain *B*. *subtilis* strains carrying different alleles of *gerAA* in one chromosomal copy, first the deletion of *gerA* operon was made with pNC-ilaR plasmid constructed as follows. A fragment of *citG* gene, upstream to *gerA* operon, was amplified from *B*. *subtilis* PY79 chromosomal DNA with citG-up and citG-dn primers, introducing *Hind*III and *Sph*I sites, respectively. PCR product was then cloned into pBEST501 [23], upstream to neomycin resistance cassette, generating pBEST501-citG. Next, DNA sequence downstream to *gerA* operon was amplified with ilaR-up and ilaR-dn primers, introducing *Bam*HI and *Kpn*I sites, respectively and cloned into pBEST501-citG, downstream to the neomycin cassette, generating pNC-ilaR. The resulting plasmid, after linearization, was used for *B*. *subtilis* PY79 transformation, selecting for neomycin resistance, generating BAG09. Chromosomal integration was confirmed by PCR. Next, chromosomal DNA of BAG09 was used for *B*. *subtilis* 168G transformation, generating BAG13. Deletion of *gerA* operon, equivalent to the lack of GerA GR in spores, was confirmed by spore germination tests with L-alanine as the sole germinant, as described below. The resulting strain, BAG13 was then complemented with *gerAB* and *gerAC* genes, coming from *B*. *subtilis* 168G chromosome, at *thrC* using a three-step cloning procedure. First, the promoter sequence of *gerA* operon was amplified from *B*. *subtilis* 168G chromosomal DNA, using an upstream gerAA-Eco and downstream gerAA-prom2R primers with addition of *Eco*RI and *Hind*III sites, respectively, and cloned into pDG1663 integration vector [24], generating pAG108. Second, *gerAC* gene was PCR amplified with primers gerAC-F and gerAC-R. PCR product containing *Hind*III site downstream of gerAC-F primer and an additional *Bam*HI site introduced in gerAC-R primer was cloned into pAG108, downstream to the *gerA* promoter sequence, generating pAG109. Third, *gerAB* gene was amplified with gerAB-F and gerAB-R primers, both introducing an additional *Hind*III site enabling cloning PCR product into pAG109, upstream to *gerAC* sequence, generating pAG110. The resulting vector, after linearization, was used for transformation of BAG13, selecting for erythromycin resistance, generating BAG14. Finally, BAG14 was transformed with linearized pAG100, pAG101 and pAG102, selecting for chloramphenicol resistance, generating BAG15, BAG16 and BAG17, respectively. The same genetic manipulations, described above, were performed for *B*. *subtilis* 168F. The final strains, BAG31, BAG32 and BAG33 carry *gerAA* sequence present in pAG100, pAG101 and pAG102, respectively.

To construct *B*. *subtilis* strains carrying two alleles of *gerAA*, one in the original locus, under the natural promoter and the other at *amyE*, under the *sspB* promoter, first, pAG112, pAG113 and pAG114 were constructed as follows. Full-length *sspB* promoter sequence, together with the *sspB* RBS, were PCR amplified with PsspB-F and PsspB-R primers, introducing *Kpn*I and *Hind*III sites respectively, and cloned into pDL vector [22], generating pAG111. Next, *gerAA* coding sequence was PCR amplified from *B*. *subtilis* 168G, 168F and PY79 chromosomal templates with gerAA-FH and gerAA-RH primers carrying *Hind*III sites. Both sequence variants were cloned into pAG111, downstream to P_*sspB*_ sequence, generating pAG112, pAG113 and pAG114 respectively. Resulting vectors, after linearization, were transformed into *B*. *subtilis* 168G, 168F and PY79 in different combinations, selecting for chloramphenicol resistance and generating BAG21, BAG22, BAG23, BAG24, BAG26, BAG27 and BAG29. The transformation events pointing at the strain and plasmid used for transformation, together with the resulting strain are presented in detail in Table 2. Chromosomal integration was confirmed by PCR method with the primers upstream and downstream to *amyE*.

Variants of *gerAA* at *gerA* and/or *amyE* were verified by qPCR method with HRM analysis (using HRM-F and HRM-R primers, upstream and downstream to 895–904 region of *gerAA* gene) in all of the strains described above.

The sequences of all primers listed above are gathered in S4 Table.

### Site-directed mutagenesis

To obtain three new *gerAA* variants, different from the naturally occurring ones, site-directed mutagenesis of pAG101 vector was performed. First, full-length pAG101 was PCR amplified using the following pairs of primers: mut302Ala-F and mut302Ala-R, mut302Gly-F and mut302Gly-R, mut_299T302P_F and mut_299T302P_R, generating pAG103, pAG105 and pAG115, respectively. Prior to *E*. *coli* DH5α transformation with the PCR products, template vector was digested in each sample with *Dpn*I. Resulting vectors, as confirmed by DNA sequencing, carry 299A/302A, 299A/302G and 299T/302P *gerAA* variant, and were used, after linearization, for transformation of BAG14 strain, selecting for chloramphenicol resistance, generating BAG18, BAG20 and BAG30 respectively. The sequences of the primers listed above are gathered in S4 Table.

### Spore preparation and purification

Spores were prepared in Difco sporulation medium [25] at 37^⁰^C and after 48-hour incubation time harvested, washed once with distilled water and stored at 4^⁰^C. Over the period of 5 to 10 days, spore suspensions were repeatedly centrifuged and washed with cold distilled water. After the purification, the purity of spores (presence of more than 95% of phase-bright spores in spore suspension) was verified by phase-contrast microscopy. At least two independent spore preparations were performed for each strain.

### Overlay test / TZM germination test

5μl of purified spore suspensions at OD_600_ of ~ 4 were dropped on a nitrocellulose membrane and heat-activated at 80^⁰^C for 20 min. Next, the membrane was placed on blotting paper presoaked in NB medium supplemented with 1% glucose and 4 mg/ml Tzm, and incubated overnight at 30^⁰^C in aluminum foil.

### Spore germination

Spore germination analysis in L-alanine solution was performed by measuring DPA release as described previously [26]. In short, spores were heat-activated at 80^⁰^C for 20 min, cooled on ice and germinated at OD_600_ of ~ 0.5 in 200μl of solution containing 25mM HEPES pH 7.5, 50μM terbium chloride and 10mM L-alanine (or 100mM L-alanine, where noted). Germination was carried in 96-well plate at 37^⁰^C in BioTek Synergy H1 microplate reader. DPA release was monitored by measuring the fluorescence emission of the DPA-Tb^3+^ complex at 545 nm with the excitation wavelength at 270nm. The measurements were taken for 120 min in 5 min time points starting from the resuspension of spores in the nutrient germinant solution. The first measurement (time zero) was used as a blank. The percent of released DPA in each time point was calculated by referring the obtained RFU values to the RFU from the same spore samples after releasing spores’ total DPA by boiling [27]. At least two independent measurements (in triplicates) were taken for each spore preparation. The percentages of spores germinated during the experiments were also routinely checked at the end of the measurements by phase-contrast microscopy and the counts corresponded to the ones obtained from the fluorescence reads. Maximum rate of germination was calculated by dividing the highest increment of DPA released from the spores, calculated for the 5-minute intervals between the consecutive measurements during spore germination analysis, by the time of the release (in minutes). DR50 value (“DPA release 50”) is the time, in minutes, in which half-maximal amount of DPA was released from the core of the spores during spore germination assays. It comes from the curve-fitting analysis by Four Parameter Logistic Regression performed in R program [28].

### *In silico* analyses

Membrane spanning fragments of GerAA protein were predicted by TMAP (http://www.bioinformatics.nl/cgi-bin/emboss/tmap), TMPred (http://www.ch.embnet.org/software/TMPRED_form.html) and TMHHM (http://www.cbs.dtu.dk/services/TMHMM/) servers. GerAA secondary structure was predicted using Jpred4 server [29].

## Supporting information

S1 Fig GerAA secondary structure prediction. Prediction was performed using JPred v.4 Protein Secondary Structure Prediction Server [29]. Red cylinders below GerAA amino acid sequence correspond to α-helices; green arrows indicate predicted β-sheets.
(TIF)

S1 Table Single nucleotide polymorphisms discovered in 168G and 168F genomes. (PDF)

S2 Table List of loci containing >20 nt differences in sequences of 168F and 168G variants. (PDF)

S3 Table Transmembrane segments of GerAA’s amino acid chain predicted by different transmembrane fragment predicting servers. (PDF)

S4 Table List of the primers used during the study for construction of plasmids. (PDF)

## References

[1] Zeigler DR, Prágai Z, Rodriguez S, Chevreux B, Muffler A, Albert T, et al The origins of 168, W23, and other *Bacillus subtilis* legacy strains. J. Bacteriol. 2008;190: 6983–6995. doi: 10.1128/JB.00722-08 18723616 10.1128/JB.00722-08 PMC2580678

[2] Burkholder PR, Giles NH. Induced biochemical mutations in *Bacillus subtilis*. Am J Bot. 1947;34: 345–348. 20252518

[3] Srivatsan A, Han Y, Peng J, Tehranchi AK, Gibbs R, Wang JD, et al High-precision, whole-genome sequencing of laboratory strains facilitates genetic studies. PLoS Genet. 2008; doi: 10.1371/journal.pgen.1000139 18670626 10.1371/journal.pgen.1000139 PMC2474695

[4] Kunst F, Ogasawara N, Moszer I, Albertini AM, Alloni G, Azevedo V, et al The complete genome sequence of the gram-positive bacterium *Bacillus subtilis*. Nature. 1997;390: 249–256. doi: 10.1038/36786 9384377 10.1038/36786

[5] Setlow P. Spore germination. Curr Opin Microbiol. 2003;6: 550–556. 14662349 10.1016/j.mib.2003.10.001

[6] Setlow P. Germination of spores of *Bacillus* species: what we know and do not know. J Bacteriol. 2014;196: 1297–1305. doi: 10.1128/JB.01455-13 24488313 10.1128/JB.01455-13 PMC3993344

[7] Moir A, Kemp EH, Robinson C, Corfe BM. The genetic analysis of bacterial spore germination. J Appl Bacteriol. 1994;77: 9S–14S. 7989261

[8] Hudson KD, Corfe BM, Kemp EH, Feavers IM, Coote PJ, Moir A. Localization of GerAA and GerAC germination proteins in the *Bacillus subtilis* spore. J Bacteriol. 2001;183: 4317–4322. doi: 10.1128/JB.183.14.4317-4322.2001 11418573 10.1128/JB.183.14.4317-4322.2001 PMC95322

[9] Paidhungat M., Setlow P. Role of Ger proteins in nutrient and nonnutrient triggering of spore germination in *Bacillus subtilis*. J Bacteriol. 2000;182: 2513–2519. 10762253 10.1128/jb.182.9.2513-2519.2000 PMC111315

[10] Alzahrani OM, Moir A. Spore germination and germinant receptor genes in wild strains of *Bacillus subtilis*. J Appl Microbiol. 2014;117: 741–749. doi: 10.1111/jam.12566 24916603 10.1111/jam.12566

[11] Wilson MJ, Carlson PE, Janes BK, Hanna PC. Membrane topology of the *Bacillus anthracis* GerH germinant receptor proteins. J Bacteriol. 2012;194: 1369–1377. doi: 10.1128/JB.06538-11 22178966 10.1128/JB.06538-11 PMC3294866

[12] Korza G, Setlow P. Topology and accessibility of germination proteins in the *Bacillus subtilis* spore inner membrane. J Bacteriol. 2013;195: 1484–1491. doi: 10.1128/JB.02262-12 23335419 10.1128/JB.02262-12 PMC3624538

[13] Mongkolthanaruk W, Cooper GR, Mawer JSP, Allan RN, Moir A. Effect of amino acid substitutions in the GerAA protein on the function of the alanine-responsive germinant receptor of *Bacillus subtilis* spores. J Bacteriol. 2011;193: 2268–2275. doi: 10.1128/JB.01398-10 21378197 10.1128/JB.01398-10 PMC3133101

[14] Biaudet V, Samson F, Bessières P. Micado a network-oriented database for microbial genomes. Comput Appl Biosci. 1997;13: 431–438. 9283758 10.1093/bioinformatics/13.4.431

[15] Krawczyk AO, de Jong A, Omony J, Holsappel S, Wells-Bennik MH, Kuipers OP, et al Spore heat activation requirements and germination responses correlate with sequences of germinant receptors and with the presence of a specific *spoVA*^2mob^ operon in foodborne strains of *Bacillus subtilis*. Appl Environ Microbiol. 2017; doi: 10.1128/AEM.03122-16 28130296 10.1128/AEM.03122-16 PMC5359491

[16] Pace CN, Scholtz JM. A helix propensity scale based on experimental studies of peptides and proteins. Biophys J. 1998;75: 422–427. 9649402 10.1016/s0006-3495(98)77529-0 PMC1299714

[17] Cabrera-Martinez RM, Tovar-Rojo F, Vepachedu VR, Setlow P. Effects of overexpression of nutrient receptors on germination of spores of *Bacillus subtilis*. J Bacteriol. 2003;185: 2457–2464. doi: 10.1128/JB.185.8.2457-2464.2003 12670969 10.1128/JB.185.8.2457-2464.2003 PMC152624

[18] Paidhungat M, Setlow P. Localization of a germinant receptor protein (GerBA) to the inner membrane of *Bacillus subtilis* spores. J Bacteriol. 2001;183: 3982–3990. doi: 10.1128/JB.183.13.3982-3990.2001 11395462 10.1128/JB.183.13.3982-3990.2001 PMC95281

[19] Igarashi T, Setlow P. Interaction between individual protein components of the GerA and GerB nutrient receptors that trigger germination of *Bacillus subtilis* spores. J Bacteriol. 2005;187: 2513–2518. doi: 10.1128/JB.187.7.2513-2518.2005 15774895 10.1128/JB.187.7.2513-2518.2005 PMC1065238

[20] Stewart KA, Setlow P. Numbers of individual nutrient germinant receptors and other germination proteins in spores of *Bacillus subtilis*. J Bacteriol. 2013;195: 3575–3582. doi: 10.1128/JB.00377-13 23749970 10.1128/JB.00377-13 PMC3754565

[21] Darling ACE, Mau B, Blattner FR, Perna NT. Mauve: multiple alignment of conserved genomic sequence with rearrangements. Genome res. 2004;14: 1394–1403. doi: 10.1101/gr.2289704 15231754 10.1101/gr.2289704 PMC442156

[22] Yuan G, Wong S-L. Regulation of *groE* expression in *Bacillus subtilis*: the involvement of the σ^A^-like promoter and the roles of the inverted repeat sequences (CIRCE). J Bacteriol. 1995;177: 5427–5433. 7559325 10.1128/jb.177.19.5427-5433.1995 PMC177347

[23] Itaya M, Kondo K, Tanaka T. A neomycin resistance gene cassette selectable in a single copy state in the *Bacillus subtilis* chromosome. Nucleic Acids Res. 1989;17: 4410 2500645 10.1093/nar/17.11.4410 PMC317980

[24] Guérout-Fleury A, Frandsen N, Stragier P. Plasmids for ectopic integration in *Bacillus subtilis*. Gene. 1996;180: 57–61. 8973347 10.1016/s0378-1119(96)00404-0

[25] Schaeffer P, Millet J, Aubert JP. Catabolic repression of bacterial sporulation. Proc Natl Acad Sci USA 1965;54: 704–711. 4956288 10.1073/pnas.54.3.704 PMC219731

[26] Yi X, Setlow P. Studies of the commitment step in the germination of spores of *Bacillus* species. J Bacteriol. 2010;192: 3424–3433. doi: 10.1128/JB.00326-10 20435722 10.1128/JB.00326-10 PMC2897682

[27] Nicholson WL, Setlow P. Sporulation, germination and outgrowth In: Harwood CR, Cutting SM, editors. Molecular biological methods for *Bacillus*. Chichester: John Wiley & Sons; 1990 pp. 391–450.

[28] R Development Core Team (2008). R: A language and environment for statistical computing R Foundation for Statistical Computing, Vienna, Austria ISBN 3-900051-07-0, URL http://www.R-project.org.

[29] Drozdetskiy A, Cole C, Procter J, Barton GJ. JPred4: a protein secondary structure prediction server. Nucl Acids Res. 2015; doi: 10.1093/nar/gkv332 25883141 10.1093/nar/gkv332 PMC4489285

