# Supplementary material for: Positions 299 and 302 of the GerAA subunit are important for function of the GerA spore germination receptor in Bacillus subtilis
Source: PLoS One. 2018 Jun 1;13(6):e0198561. doi: 10.1371/journal.pone.0198561 (PMC5983566; doi:10.1371/journal.pone.0198561)
Supplement: S1 Table — (PDF) [file pone.0198561.s002.pdf]

Position	Product
161480	16S rRNA
166772	16S rRNA
961826	Aliphatic sulfonate ABC transporter ATP-binding protein TauB
2271518	hypothetical protein
2271536	hypothetical protein
3010794	argininosuccinate ligase ArgH
3391688	spore germination protein GerAA
3391697	spore germination protein GerAA
4005705	purine nucleoside transporter NupG
4095823	hypothetical protein RoxA – ribosomal protein L16 Arg18 hydroxylase
