# Supplementary material for: Positions 299 and 302 of the GerAA subunit are important for function of the GerA spore germination receptor in Bacillus subtilis
Source: PLoS One. 2018 Jun 1;13(6):e0198561. doi: 10.1371/journal.pone.0198561 (PMC5983566; doi:10.1371/journal.pone.0198561)
Supplement: S2 Table — (PDF) [file pone.0198561.s003.pdf]

**Locus**	**Product**
rrnA-16S	16S rRNA
rrnA-23S	23S rRNA
amyA	alpha-amylase
ppsA/dacC	plipstatin synthase subunit A/D-alanyl-D-alanine carboxypeptidase, intergenic region
sunA/sunI	Sublancin precursos/protein of immunity to sublancin, SPBc2 prophage intregenic region
uvrX	UV-damage repair protein
zwf/gndA	glucose-6-phosphate 1-dehydrogenase/NADP+-dependent 6-P-gluconate dehydrogenase, intergenic region
sdpR/opuCD	transcriptional regulator (Ars family)/glycine/betaine/carnitine/choline/ choline sulfate ABC transporter (permease), intergenic region
ykuV/rok	thiol-disulfide oxidoreductase/repressor of ComK, intergenic region
yjcZ	sporulation protein, putative prophage protein
phrA/yjpA	secreted inhibitor of the activity of phosphatase RapA/putative enzyme, intergenic region
ggaB	minor teichoic acid biosynthesis protein
yydD	hypothetical protein
cpdA/rlmH	metallophosphoesterase/23S rRNA (pseudouridine(1915)-N(3)) methyltransferase
yybN	hypothetical protein
yybL	hypothetical protein
yybK	hypothetical protein
