# Supplementary material for: Positions 299 and 302 of the GerAA subunit are important for function of the GerA spore germination receptor in Bacillus subtilis
Source: PLoS One. 2018 Jun 1;13(6):e0198561. doi: 10.1371/journal.pone.0198561 (PMC5983566; doi:10.1371/journal.pone.0198561)
Supplement: S3 Table — (PDF) [file pone.0198561.s004.pdf]

	Transmembrane segment 1	Transmembrane segment 2	Transmembrane segment 3	Transmembrane segment 3
TMAP	238-264	280-308	360-388	402-430
TMPrerd	242-265	284-300	373-392	407-429
TMHMM	242-264	279-301	347-396	406-428
