# Supplementary material for: Positions 299 and 302 of the GerAA subunit are important for function of the GerA spore germination receptor in Bacillus subtilis
Source: PLoS One. 2018 Jun 1;13(6):e0198561. doi: 10.1371/journal.pone.0198561 (PMC5983566; doi:10.1371/journal.pone.0198561)
Supplement: S4 Table — (PDF) [file pone.0198561.s005.pdf]

Name	Sequence
gerAA-Eco	ACGCGGGAATTCTTTGTATATTTG
gerAA-Bam	AGGGATCCAGTAGTCAGGCGTATTAG
citG-up	TTATAAGCTTGCTACCACTTCGTTCATG
citG-dn	TTAAGCATGCCATTAGATCATTTGAACAGC
ilaR-up	ATATAGGATCCAGCCGCCTAATTCACGAG
ilaR-dn	ATATGGTACCGGCTTGCTGTGGAACTGTC
gerAA-prom2R	TCCAAAAGCTTAGTAGAGGTTATC
gerAC-F	GCTGGGACAGTGAGAATATCGAG
gerAC-R	AAGGGGGATCCGAAGCTGGAGTC
gerAB-F	AAAAGCTTAAAAGAGGTGAATAATCCAAATGAGC
gerAB-R	CTAAGCTTAATTCCTCGATATTCTCAC
PsspB-F	TCA AGA GGT ACC ACA CAA TTC TC
PsspB-R	TGG TTA GCC AAG CTT AAA ATC TCC
gerAA-FH	GAG GTG ACA AGC TTG GAA CAA ACA GAG
gerAA-RH	AGT GGT AAG CTT TCG CGG CAA TG
HRM-F	GCTTTGCTTCTATCTTTATCACCTTG
HRM-R	CGGCAGCAGCCCCTGATG
mut302Ala-F	CGATTTATATAGCACTTGTTGCCTTCCATCAGGGGCTGCTG
mut302Ala-R	CAGCAGCCCCTGATGGAAGGCAACAAGTGCTATATAAATCG
mut302Gly-F	GATTTATATAGCACTTGTTGGATTCCATCAGGGGCTGCTG
mut302Gly-R	CAGCAGCCCCTGATGGAATCCAACAAGTGCTATATAAATC
mut_299T302P_F	GTCATCGATTTATATAACGCTTGTTCCCTTCCATCAG
mut_299T302P_R	CTGATGGAAGGGAACAAGCGTTATATAAATCGATGAC
